# Advancing the functional utility of PAR-CLIP by quantifying background binding to mRNAs and lncRNAs

**DOI:** 10.1186/gb-2014-15-1-r2

**Published:** 2014-01-07

**Authors:** Matthew B Friedersdorf, Jack D Keene

**Affiliations:** 1 Department of Molecular Genetics & Microbiology, Duke University Medical Center, Durham, NC, USA

## Abstract

**Background:**

Sequence specific RNA binding proteins are important regulators of gene expression. Several related crosslinking-based, high-throughput sequencing methods, including PAR-CLIP, have recently been developed to determine direct binding sites of global protein-RNA interactions. However, no studies have quantitatively addressed the contribution of background binding to datasets produced by these methods.

**Results:**

We measured non-specific RNA background in PAR-CLIP data, demonstrating that covalently crosslinked background binding is common, reproducible and apparently universal among laboratories. We show that quantitative determination of background is essential for identifying targets of most RNA-binding proteins and can substantially improve motif analysis. We also demonstrate that by applying background correction to an RNA binding protein of unknown binding specificity, Caprin1, we can identify a previously unrecognized RNA recognition element not otherwise apparent in a PAR-CLIP study.

**Conclusions:**

Empirical background measurements of global RNA-protein crosslinking are a necessary addendum to other experimental controls, such as performing replicates, because covalently crosslinked background signals are reproducible and otherwise unavoidable. Recognizing and quantifying the contribution of background extends the utility of PAR-CLIP and can improve mechanistic understanding of protein-RNA specificity, protein-RNA affinity and protein-RNA association dynamics.

## Background

RNA binding proteins (RBPs) control many important and interconnected steps in posttranscriptional gene expression, including co-transcriptional regulation, epigenetics, pre-RNA processing, mRNA splicing, nuclear export, quality control, subcellular localization, stability and translation [1]. Furthermore, RBPs can coordinate production of functionally related proteins through posttranscriptional operons/regulons [2,3]. RBPs achieve these complex regulatory functions of interconnection and coordination by binding to specific RNA recognition elements. The ultimate regulatory fate of a transcript is defined by its unique “RNP code”, a medley of RNA recognition elements in a single RNA that is combinatorially regulated by a network of RBPs [2,4,5]. Moreover, RNA-protein interactions play critical roles in both normal and diseased states [6-10].

To identify global *in vivo* RNA-protein interactions, numerous methods have been applied including immunopurification (IP) of RBPs followed by microarray analysis (RIP-Chip) or sequencing (RIP-seq). Essential to the identification of bound RNAs by RIP-Chip is the empirical measurement of background binding by using a control IP with isotype matched IgG or other mock proteins [11,12]. The determination of background has proved highly valuable by allowing widespread and reproducible use of standardized RIP-Chip procedures [13]. Furthermore, background measurements have been used as a reference for quantitative determination of bound transcripts and to calculate fold changes following cellular perturbations, such as T cell activation [14].

To identify individual bindings sites of RBPs within a RNA target, several distinct but related techniques have been developed based on covalent UV crosslinking followed by IP and high-throughput sequencing (CLIP) (reviewed in [15]). Most CLIP-related procedures assume that background binding is biochemically eliminated through rigorous and stringent washes given that non-covalent bonds have been replaced by UV-induced covalent bonds. This approach to dealing with background binding has produced notable difficulties that have limited the widespread and standardized use of these techniques, evidence for which can be seen in the number of new, distinct crosslinking techniques that have been devised of necessity (reviewed in [16,17]). For example, PAR-CLIP is a useful and novel crosslinking approach devised by Tuschl and coworkers that addresses the issue of indirect targets arising from non-crosslinked noise, and discriminates indirect from direct RNA-protein interactions by identifying diagnostic conversions resulting from covalent adducts at sites of crosslinking of the RBP to specific nucleotides [18]. However, none of the CLIP-related studies has quantitatively addressed the issue of background generated by adventitious covalent crosslinking of RNAs to proteins that are not the RBP of interest.

In this study, we demonstrate that covalently crosslinked background binding during the PAR-CLIP procedure is common, reproducible and likely universal to crosslinking procedures, and that this can have serious implications for understanding protein-RNA specificity as well as protein-RNA affinity. Given the relative inefficiency of UV crosslinking procedures in general, characteristic sequence biases and the exquisite sensitivity of high-throughput sequencing, false binding targets should be expected [12,19-22]. We find that low affinity RBPs are especially affected by false crosslinking events that can limit the ability of global analysis to determine authentic binding sites. These problems are especially acute when attempting to study the effects of mutations in RNA-binding domains on underlying RNA recognition mechanisms or, to discern dynamic changes in RNA targets following induced perturbations or varied growth conditions. In this study, we show that by quantifying and accounting for background, as is standard in RIP-Chip protocols, we are able to both improve the specificity of PAR-CLIP target identification and remove erroneous and misleading results. Among several examples of the utility of these improvements, we reveal false binding sites in *XIST* and *MALAT1* lncRNAs and use a previously published PAR-CLIP dataset to identify a novel A-rich RNA motif in the RBP, Caprin1.

## Results and discussion

### PAR-CLIP background contains many uniquely mapping sequence reads

**Figure 1:**
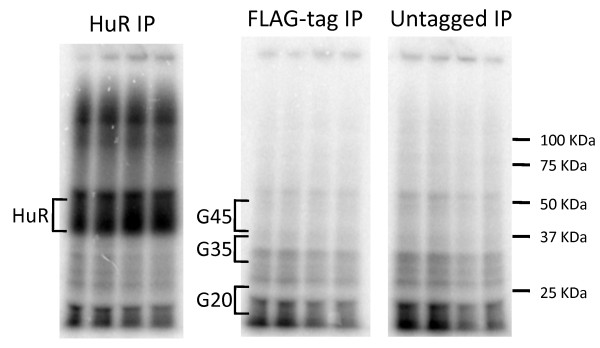
**Control immunoprecipitations of PAR-CLIP gels show significant background RNA.** Phosphorimages of SDS acrylamide gels following the PAR-CLIP protocol show radiolabeled RNPs from HEK293 cell lysates. The IP from each lysate contains four replicates. Brackets indicate extracted regions of the HuR RBP and controls using FLAG-GFP (tagged green fluorescent protein as G20, G35, G45) and no tagged protein.

To define background binding events in PAR-CLIP data we performed the standard PAR-CLIP protocol on lysates expressing a commonly used non-RBP control, FLAG-GFP [18]. After FLAG-tag immunopurification of UV 365 nm irradiated lysates prepared from cells supplemented with 4-thiouridine (4SU), RNA was partially digested with RNase T1, radiolabeled and separated by SDS-PAGE. As in other published studies, the phosphorimage from the FLAG-tagged control IP contained radioactive signal throughout the lane, mostly as a smear but with a number of clearly identifiable discrete bands (Figure 1). When compared to a positive control RBP, HuR, the intense bands migrating approximately with wild-type HuR RNPs were absent from the control IP but the smears and discrete bands from the control were also clearly visible in the HuR lanes. These smears and discrete control bands are frequently observed in PAR-CLIP and related crosslinking procedures [18,23]. Furthermore these smears and discrete background bands were also present in lanes from lysates that did not contain a FLAG-tagged protein (untagged IP in Figure 1), suggesting that this radioactive signal represents non-specific background binding that is not due to the FLAG-tag.

To identify which RNAs were present in the background we cut out three regions of the gel migrating at different sizes, roughly around 45 KDa, 35 KDa and 20 KDa (referred to as G45, G35 and G20). We prepared sequencing libraries from the three slices and submitted the samples for high-throughput sequencing alongside a library from the HuR band. Each of the sequencing runs produced over 180 million reads which after processing and mapping contained between 6.2 and 38.6 million uniquely mapping reads (Table 1). This range in number of reads for the background samples is similar to reads from HuR PAR-CLIP experiments sequenced at the same time. However, the number of unique binding sites, defined by genomic intervals with overlapping reads, ranged from 259,337 for G35 to 706,883 for G20 which is anywhere from 3 to 20 times smaller than the unique binding sites HuR replicates. Thus, large numbers of reads are abundantly detectable from background gel slices; and while they map to fewer unique binding sites than the high affinity HuR RBP this is expected, since HuR reads would logically contain a combination of both true signal and background reads.

**Table 1:** High-throughput sequencing detected reads from PAR-CLIP background samples are abundant

	**Total reads (PARalyzer utilized)**	**Unique sites (Locations)**
G20	38,644,636	706,883
G35	6,187,300	259,337
G45	7,539,669	379,718
HuR.rep1	9,525,625	2,411,483
HuR.rep2	32,111,810	4,024,972
Total	35,924,998	2,857,291

Total PARalyzer Utilized Reads from three background samples were derived from the G20, G35 and G45 slices, two HuR replicates and a Total sample from 4SU-incorprated, UV 365 nm cross-linked and RNase T1 digested lysates that have been depleted for rRNA. Unique Locations are genomic sites derived after accounting for overlapping reads.

### Background reads contain T > C conversions

**Figure 2:**
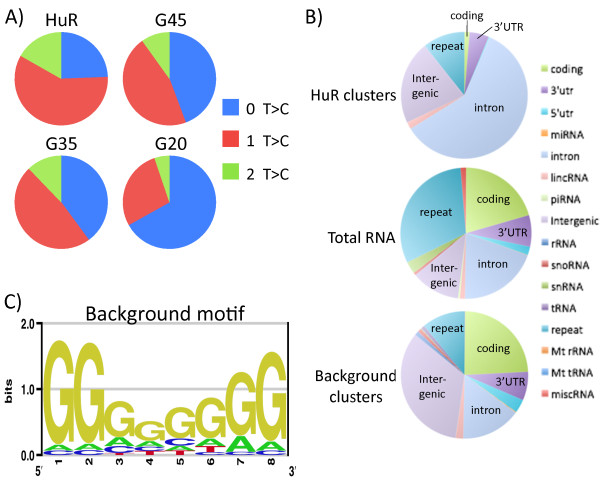
**PAR-CLIP background reads contain many T-to-C conversions that have G-rich motifs. A)** Pie charts of all PARalyzer utilized reads from HuR PAR-CLIP and the three background gel slices (G45, G35 and G20) that contain T-to-C conversions, suggesting crosslinked events. PARalyzer utilized reads are defined as sequences containing 0, 1 or 2 T-to-C conversions that map uniquely to the human reference genome. **B)** Pie charts of genomic location where HuR, total RNA and background clusters map to in human reference genome. Background clusters are union of all three background samples. Total RNA was prepared from 4SU-containing, crosslinked lysates that were partially digested with RNase T1. **C)** Motif logo of top 25 occurring 8mer motifs by union of all three background samples. The G-rich motif is significantly enriched compared to shuffled sequences preserving di-nucleotide frequencies of the library (p-value = 7.04 × 10^-8^).

PAR-CLIP takes advantage of diagnostic T-to-C conversions that occur during reverse transcription as a result of 4-thiouridine (4SU) being covalently crosslinked to protein, thus enabling direct protein-RNA interactions to be distinguished from indirect non-crosslinked interactions [18]. To test whether the background reads were due to direct or indirect interactions, we looked for the presence of T-to-C conversions in the reads for each of the libraries. The bowtie aligned reads prior to PARalyzer filtering contained many more T-to-C mismatches than any of the other possible types of mismatches, as is typical of other PAR-CLIP studies (Additional file 1: Figure S1). After using PARalyzer to filter these reads we compared the percent of PARalyzer utilized reads that contained T-to-C conversions for each of the GFP gel slices to those of HuR. In the case of HuR ~75% of reads contained a T-to-C conversion while the GFP slices contained up to 60% T-to-C conversions (Figure 2A). The percent of converted reads for HuR is well within the 50-80% values previously reported for other crosslinked RBPs [18]. Surprisingly, two of the three background slices (G35 and G45) were also in this range with over 50% T-to-C conversions and the third slice (G20), with 33%, was well over the reported *in vitro* conversion rates of 10-20% for non-crosslinked 4SU-containing RNA [18]. Furthermore, many of the background binding sites that contained evidence of multiple reads also had multiple T-to-C conversion locations. This suggests that these background conversions are mostly derived from direct protein-RNA interactions and are much more abundant than non-crosslinked *in vitro* conversions. Furthermore, this indicates that background cannot be completely removed from the data by only accounting for T-to-C conversions or by methods that reduce background by addressing only non-crosslinked RNAs, such as using transfer to nitrocellulose.

### Background reads tend to be G-rich and represent cellular RNAs

To determine if the background reads were mostly from a cellular source or were being introduced at one of the other steps, such as adapter ligation, we compared the reads mapping to annotated genomic regions versus those represented in total RNA. To control for methodological biases that may influence mapping of PAR-CLIP reads we modified the PAR-CLIP protocol for isolation of total RNA. To do this we made lysates from cells treated with 4SU and irradiated at UV 365 nm, then the lysates were proteinase K digested followed by rRNA depletion with RiboZero and finally the RNA was partially digested with RNase T1. The library preparation, sequencing and mapping parameters were identical for PAR-CLIP and for the total RNA. We observed that the percent of background PAR-CLIP reads mapping to coding, 3’utr, 5’utr, intron and lincRNA were similar to the values for total RNA reads (Figure 2B). The only significant differences between PAR-CLIP background reads and total RNA reads were that background reads mapped to repeat regions less frequently and to intergenic regions more frequently. However, when comparing background reads to reads from HuR, each mapped to drastically different regions. This indicates that the background reads are from a cellular RNA source and not simply the result of sequencing artifacts.

Next, to identify sequences and motifs that were enriched in background reads we used a kmer approach to build motifs from the union of all three GFP background libraries. The highest enriched motifs were extremely G-rich, including 24 of the top 25 most abundant 8mer sequences. After guanosine the next most common nucleoside in these motifs was adenosine followed by cytidine and uridine (Figure 2C). The enrichment of guanosine in these motifs is surprising considering the use of RNase T1, which cleaves at single stranded guanosines, furthermore the relative scarcity of uridines in the motifs is also surprising despite the use of 4SU as a specific cosslinking agent. This G-rich motif is much more abundant in background samples than in the transcriptome suggesting that this motif is the result of biases of the procedure.

### High abundance background sites are common and reproducible between different molecular weight gel slices of PAR-CLIP background

**Figure 3:**
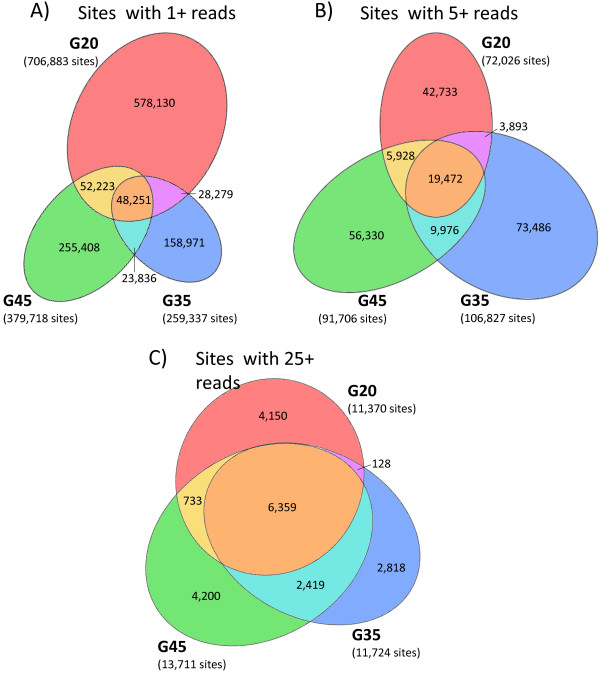
**High abundance background sites are common and reproducible between different PAR-CLIP background gel slices.** Area-proportional elliptical Venn diagrams of reads from three background samples isolated from different sizes on SDS-PAGE gel. **A)** All sites with one or more reads displayed. **B)** Sites with five or more reads displayed. **C)** Sites with 25 of more reads displayed.

Since background was taken from three gel slices that migrated at different molecular weights on an SDS-PAGE gel, it would be expected that background reads from each slice would represent different RNAs. We examined reads from the three background gel slices for overlapping sites and saw that a large majority of the sites were unique to each gel slice (Figure 3A). However, the amount of overlapping sites between the three backgrounds steadily increased when considering sites with larger read depths (Figure 3B, C). For sites with a read depth of at least 25 in each of the libraries, 55.9% of the G20 sites were found in the other two libraries while 54.2% and 46.4% of the G35 and G45 sites respectively were found in the other two libraries (Figure 3C). The percent of overlaps were even higher for reads present in at least one of the other libraries with 63.5% of the G20 sites found in at least one other library and 75.9% and 69.4% of the G35 and G45 sites found in at least one other library, respectively. This extraordinary amount of overlap between background binding sites demonstrates that background reads, especially high abundance ones, can be isolated from different band sizes on the gel and that these high abundance sites are reproducible between samples with R^2^ values ranging from 0.65 to 0.7 (Additional file 2: Figure S2).

### Background binding sites are prevalent in PAR-CLIPs of weak affinity RBPs

To compare the background PAR-CLIP binding sites to the binding sites of HuR PAR-CLIP, we focused on the reads at three representative sites, a non-coding RNA site in *MALAT1*, a coding sequence site and the 3’ UTR of the *ELAVL1* mRNA (the HuR gene transcript). *MALAT1* is a highly conserved, abundantly expressed non-coding RNA that is primarily located in the nucleus. Several recent global protein-RNA crosslinking studies have identified *MALAT1* as a target of Sfrs1, Tardbp and Dgcr8 RBPs [24-26]. Elavl1, also known as HuR, is an abundantly expressed member of the ELAV/Hu family of proteins that are well known to bind and autoregulate their own messages through 3’UTR binding sites [27-30].

**Figure 4:**
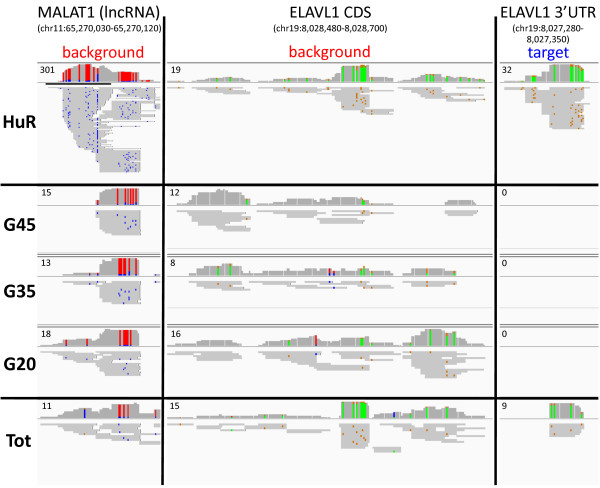
**Experimentally measured background reads distinguish commonly detected background sites from authentic binding events.** Three representative examples of genomic regions containing PAR-CLIP reads: *MATLAT1* lncRNA, *ELAVL1* coding sequence (CDS), and *ELAVL1* 3’UTR. *MALAT1* lncRNA and the *ELAVL1* CDS have significant background binding (three middle panels G20, G35, G45) while the definitive HuR RBP binding site in *ELAVL1* 3’UTR lacks any reads from background libraries but contains reads in the total (Tot) library. Grey bars represent unique sequencing reads while blue/red marks or green/tan marks represent T-to-C conversions detected on the positive or negative genomic strand, respectively. The numbers in the upper left corners are the scale of the maximum read depth for an individual nucleotide. Depictions of these binding events to the full-length *MALAT1* and *ELAVL1* transcripts are shown in Additional file 3: Figure S3 and Additional file 4: Figure S4, respectively.

While investigating HuR RBP and background PAR-CLIP data we observed many binding sites in *MALAT1* that contained overlapping reads from each of the G20, G35 and G45 background samples as well as from HuR RBP (Figure 4 and Additional file 3: Figure S3). The reads in these overlapping sites were numerous in each of the libraries, often with multiple, overlapping T-to-C conversions and similar read boundaries. We also observed a similar pattern of overlapping reads in the coding sequence site of *ELAVL1* mRNA (Figure 4 and Additional file 4: Figure S4). However, in the 3’UTR binding site of *ELAVL1* mRNA there were numerous reads with multiple T-to-C conversions in the HuR PAR-CLIP but not a single read in any of the three background samples. The difference at this 3’UTR site is not due to differences in expression or presence of the *ELAVL1* transcript in one library versus the others because we can see many reads from the background libraries in the coding sequence site of *ELAVL1*. Furthermore, we note reads present at this 3’UTR site in the total sample indicating expression. Two of the representative sites, *MALAT1* and *ELAVL1* coding sequence, which contained overlapping reads from all four libraries demonstrate that background reads can be found in RBP PAR-CLIP libraries at the same locations and that they share many characteristics, such as read boundaries and T-to-C conversions. The absence of reads from the background libraries in the *ELAVL1* 3’UTR site show that background reads can be useful for distinguishing authentic binding sites from non-specific false-binding sites. Furthermore, the similarity of characteristics at background sites to authentic binding sites, such as: length, start and stop of sites, number and locations of conversions and even read depth, suggests that purely bioinformatic-based approaches may be limited in their ability to distinguish background from true signal.

**Figure 5:**
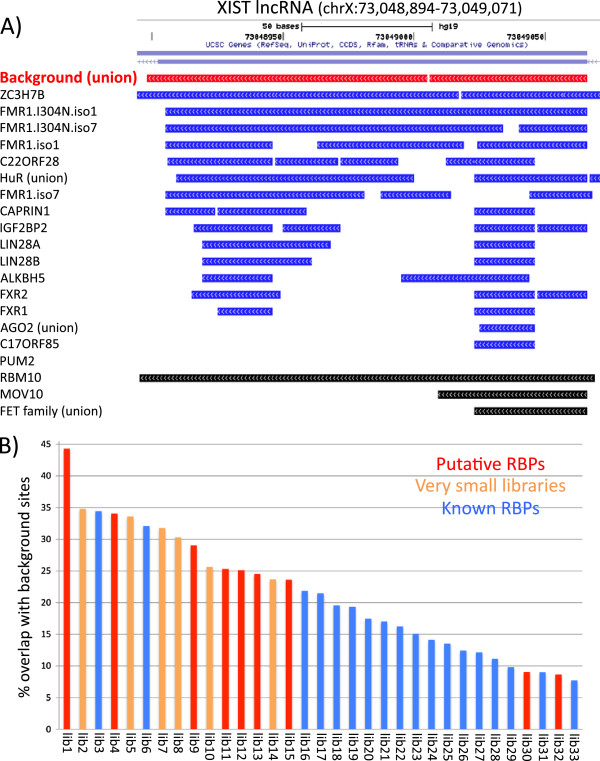
**Background binding sites are present in many published PAR-CLIP libraries especially putatively weak binding RBPs.** Previously published PAR-CLIP results from multiple studies were processed using PARalyzer and analyzed for sites that overlap with background and with each other. **A)** Genome browser window depicting overlapping sites in an exon of the *XIST* lncRNA from various PAR-CLIP studies. Red horizontal bars in the top panel indicate areas with read evidence in at least one of the three background libraries. Horizontal blue bars indicate reads from various PAR-CLIP studies, re-analyzed in this study, all with the same PARalyzer parameters. Black bars indicate PAR-CLIP studies not re-analyzed in this study but found in the doRiNA database. **B)** Bar graph depicting percent overlap of background sites with sites from 33 different PAR-CLIP experiments (see Additional file 1: Table S1 for info on RBP and study). Vertical red bars are from PAR-CLIPs of putative or “non-professional” RBPs, which are defined as previously unrecognized RBPs because they do not contain any known RNA recognition motifs or domains. Orange bars are PAR-CLIP experiments that produced very few reads, those containing roughly 10,000 or fewer reads (post-processing and mapping). Blue bars are all other PAR-CLIP experiments using established RBPs.

Since background samples can identify false-binding sites in HuR RBP libraries, we wanted to know how frequently background reads overlapped with sites from other previously published PAR-CLIP data sets. Therefore, we compiled 33 PAR-CLIP data sets from 8 different studies including a variety of RBPs. Different PAR-CLIP studies often define binding sites with different criteria, so we decided to treat each of the data sets using the same criteria. We took the raw reads from each of the indicated studies and ran them through the PARalyzer program [31] using the same standard settings to define binding sites (Additional files 5, 6, 7, 8, 9, 10, 11, 12, 13, 14, 15, 16,17, 18, 19, 20, 21, 22, 23, 24, 25, 26, 27, 28, 29, 30, 31, 32, 33, 34, 35, 36, 37, 38, 39, 40, 41, 42, 43, 44, 45, 46, 47, 48, 49, 50, 51, 52, 53, 54, 55, 56, 57, 58, 59, 60, 61, 62, 63, 64, 65 and 66). We also supplemented these data sets with some of the already processed data sets from the doRiNA database [32]. For the non-coding RNA, *XIST*[33], we observed some of the same apparent binding events in nearly all of the different RBP PAR-CLIP libraries, including many sites of identical length (Figure 5A). These PAR-CLIP data sets include a wide variety of unrelated RBPs that recognize dramatically different RNA motif elements. For example, Fmr1 recognizes two distinct motifs, WGGA and ACUK, while HuR recognizes U-rich elements [34-37]. Furthermore, these sets of PAR-CLIP data were performed in several different laboratories showing that the overlaps are not due to laboratory-specific influences. In addition to this site in *XIST*, there are numerous other examples of binding sites that we found in multiple PAR-CLIP libraries. For example *MALAT1* non-coding RNA contains numerous binding sites that are found in many of the published PAR-CLIP and iCLIP libraries (Additional file 67: Figure S5). Moreover, these broadly bound sites are frequently present in our PAR-CLIP background libraries suggesting that the binding of multiple RBPs is not due to specific interactions with each of the RBPs but rather are non-specific backgrounds common to most PAR-CLIP experiments.

To investigate how frequently PAR-CLIP data sets overlap with background we used the same re-analyzed set of PAR-CLIP data sets described above and determined the percent of reads that overlapped with reads from any one of the three background samples. We observed that over a range of 45% to less than 10% of sites in an RBP PAR-CLIP overlapped with background sites (Figure 5B and Additional file 68: Table S1). Interestingly, we noticed that the PAR-CLIP experiments with a higher percent overlap with background were frequently either from PAR-CLIP studies on putative RBPs, previously uncharacterized RBPs, or PAR-CLIP libraries that had produced very few mapped reads. This demonstrates that the degree of a particular PAR-CLIP library overlapping with background varies greatly depending on the RBP; and weak binding (or weakly crosslinked) RBPs in particular contain a larger fraction of background binding. In many cases, these RBPs are considered putative based upon the absence of a known RNA-binding motif, and have been characterized as non-professional RBPs [38,39]. Moreover, this can be exceedingly important when investigating defined mutations in professional RNA-binding domains, as well as dynamic *in vivo* binding events that may involve sequential low-affinity maturation such as occurs with cooperative binding experiments.

**Figure 6:**
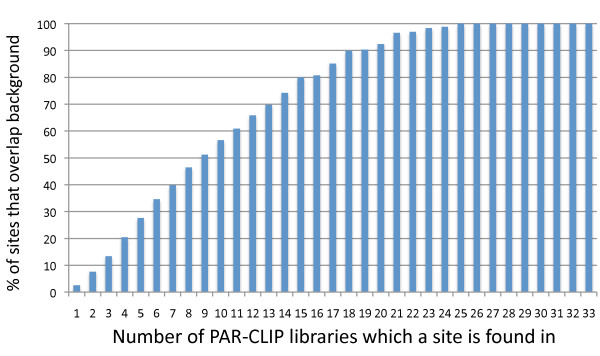
**Sites found in an increasing number of PAR-CLIP libraries increase in their percent overlap with PAR-CLIP background sites.** X-axis shows bins of PAR-CLIP identified sites appearing in exactly the indicated number of different PAR-CLIP libraries. Y-axis indicates the percent overlap for those sites with background sites.

In the example of the *XIST* exon many different PAR-CLIP experiments demonstrated binding at a single site, which was also present in background PAR-CLIPs. To investigate how frequently sites found in multiple PAR-CLIP libraries were also present in background PAR-CLIPs we binned sites by the number of libraries they appeared in and graphed it against the percent of those sites that overlapped with the union of the background libraries. We observed that sites only found in a few PAR-CLIP libraries overlapped with background sites with low frequency (~2.5% for unique sites) but as the number of libraries a site was found in increased, so did the percent of those sites overlapping with background (Figure 6). Eventually, at sites found in 25 libraries or more, 100% of the sites were also present in the background. This demonstrates that background binding is universally found in many different PAR-CLIP libraries and that sites with reads from multiple libraries are often present in those libraries because they are in essence, built into the system.

**Figure 7:**
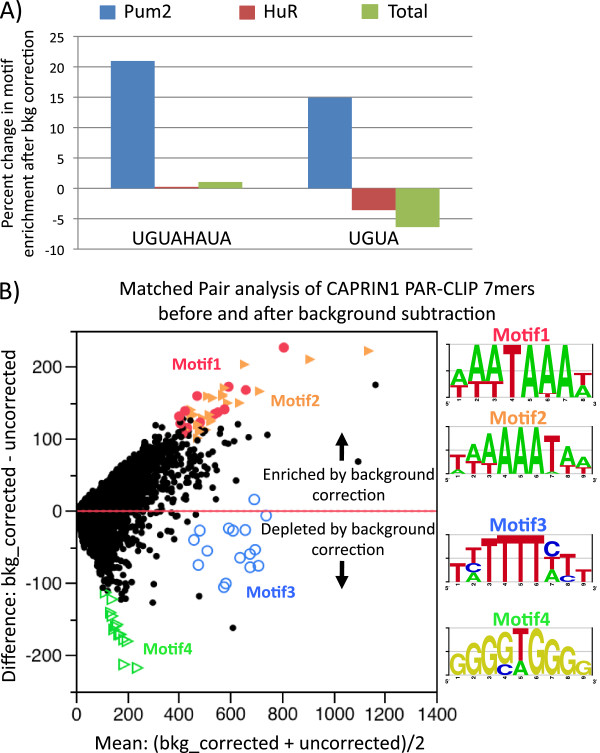
**Accounting for background binding dramatically improves motif finding from PAR-CLIP data. A)** Percent change in motif enrichment after background correction for full Pum motif (UGUAHAUA) and half Pum motif (UGUA). Blue bars indicate improvement for Pum library after background correction. HuR (red bars) and Total (green bars) are controls that do not specifically associate with Pum motifs. **B)** Matched-pair analysis of 7mers from uncorrected Caprin1 sites versus 7mers from background corrected Caprin1 sites (normalized for library size). Motif 1 and Motif 2 are enriched by background correction and both are A-rich with motif 1 containing poly (A) signal sequences. Motif 3 is U-rich and moderately depleted by background correction while Motif 4 is G-rich and dramatically depleted.

Interestingly, we also observed that the more abundant sites in a PAR-CLIP library overlapped with background much more frequently than lower abundance PAR-CLIP sites (Additional file 69: Figure S6). This is similar to our previous observation in Figure 3 that reads from the three different background gel slices overlap most strongly among the most abundant binding sites. This implies that many of the abundant reads from *any* PAR-CLIP library may represent RNAs that are enriched by biases in the protocol, such as UV nucleotide preferences or ligation site sequence biases, rather than being the most strongly bound sequences. The strong overlap of abundant binding sites with background also suggests that at least some of the background is derived from abundant RNAs in the cell. Therefore it is also possible that some of the strong overlap we observe is a result of highly abundant “true” RBP RNA targets that show up as false positives in background samples. However, it should be noted that improvements in motif discovery by background correction, as discussed below, was more robust for high abundance sites than for low abundance sites (Figure 7B and Additional file 70: Figure S7). This suggests that simply taking the most abundant sites in a PAR-CLIP experiment as targets might increase background and could result in extremely biased results.

### Background correction markedly improves motif finding

We have shown above that background binding is a common and reproducible feature of PAR-CLIP, and it is essential to assess its utility in data interpretation. To this end, we investigated how background correction can be used to improve motif finding. Therefore, we analyzed Pum2, a member of the Puf family of RBPs that are widely known for their highly specific and evolutionarily conserved recognition of RNA elements [40,41]. In particular, mammalian Pum proteins are known to bind to mRNAs containing one or more UGUAHAUA sequences [42-44]. Hafner and colleagues validated the specificity of PAR-CLIP by showing that Pum2 PAR-CLIP can enrich for Pum motifs (UGUAHAUA), especially half-site Pum motifs (UGUA), and revealed positional information of binding [18]. To correct for background binding, we eliminated sites from Pum2 PAR-CLIP that overlapped by one or more nucleotides with sites from at least one of the three PAR-CLIP background samples. Background correction dramatically increased the percent of sites that contained either Pum motifs or half-site Pum motifs, with increases in specificity of up to 1.2 fold (Figure 7A). When compared to HuR, an RBP that is not known to bind to Pum motifs, background correction did not show any noticeable improvement in enrichment of the full motif and a negative enrichment of the half motif. This lack of Pum motif enrichment was also observed when background correction was applied to the total library. An increase of 20% in specificity can be highly significant, even crucial, for assigning confidence values to motifs, most of which are not as definitive as the Puf motif. For example, computational searches for motifs are most precise when the motif itself is definitive. Overall, our data demonstrate that background correction significantly improves PAR-CLIP specificity by eliminating non-specific sequences (i.e. those not containing Pum motifs) in preference over sequences that do contain Pum motifs.

While background correction significantly improved the specificity for identifying Pum motifs, it was already shown that PAR-CLIP, prior to background correction, could also identify these highly specific motifs. Thus, to further test our approach, we examined whether background correction can offer significant improvement in identifying a truly novel motif. To do this we attempted to identify a candidate motif from published PAR-CLIP data of Caprin1, a known RBP that has been shown by using PAR-CLIP to bind to both coding regions and 3’UTRs but for which no motif has been identified [38,45,46]. To identify possible motifs for Caprin1, we counted the abundance of 7mer sequences within the PAR-CLIP identified sites (Figure 7B). Among the most common 7mers were two motifs; one motif was U-rich with occasional adenines and cytosines, while the other motif was A-rich with occasional uridines. A third, less common, G-rich motif was also observed. We then performed a matched pairs analysis on the 7mers before and after background correction to identify sequences that were enriched or depleted by background correction. The matched pair analysis allowed us to investigate the more abundant motifs, plotted along the x-axis, on a continuum without setting arbitrary cutoffs. We observed that background correction strongly enriched for the A-rich motifs while moderately depleting the U-rich motifs and strongly depleting the G-rich motifs (Figure 7B). As expected, the depleted G-rich motifs were similar to the G-rich motifs that were found most frequently in the background PAR-CLIPs. Among the strongly enriched A-rich motifs were a subset of sequences that contained canonical polyA signal sequences. The similarity of this motif to the polyA signal sequence led us to investigate the enrichment of Caprin1 RNA-binding sites at the end of transcripts, and we found that 3’ termini were enriched in background corrected Caprin1 PAR-CLIP sites. This suggests that recognition of motifs containing polyA signal sequences by Caprin1 maybe functional and may indicate a role for Caprin1 in polyadenylation. Our data suggests that we have identified a candidate A-rich motif, which includes canonical polyA signal sequences, for Caprin1. This motif needs to be confirmed biochemically, but it was not obvious from the uncorrected PAR-CLIP data (Additional file 70: Figure S7). Therefore, by using background correction we were able to distinctly identify novel candidate motifs for an RBP, demonstrating the utility of our approach.

### Background correction can remove misleading results from PAR-CLIP data

**Figure 8:**
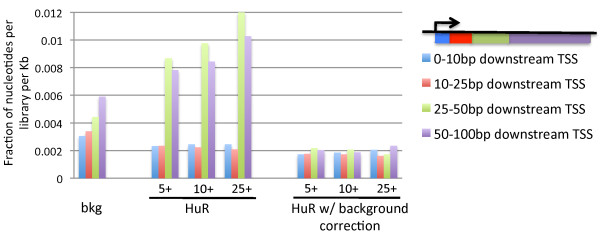
**Background correction removes misleading enrichment of HuR binding in region downstream of transcription start sites.** Reads from each library were normalized for number of nucleotides per library and length of region (fraction of nucleotides per library per Kb). Background reads were derived from union of all three backgrounds. Numbers 5+, 10+ and 25+ indicate HuR clusters with 5, 10, or 25 or more reads, per cluster. Regions 25-50 bp downstream of transcription start sites (green bars) and 50-100 bp downstream (purple bars) appear enriched prior to background correction; however, they were no longer enriched after correction.

In addition to identifying bound sequences, PAR-CLIP is also able to provide positional information about binding [18]. To investigate whether background correction can improve this aspect of PAR-CLIP data, we compared it with our wild-type HuR PAR-CLIP dataset. Within the HuR dataset, prior to background correction, PARalyzer-identified binding sites were enriched in regions 25-100 bp downstream of transcription start sites (Figure 8). This curious binding pattern has not previously been reported for HuR and seemed to suggest a novel unrecognized mode of regulation by HuR. However, upon background correction these sites downstream of transcription start sites were no longer enriched, thus suggesting that binding downstream of the transcription start sites was due to non-specific false positive events. This analysis demonstrates that background correction can improve percent signal of samples as seen in the improvement of motifs (Figure 7A), but can also remove misleading results such as indications of false binding site enrichment.

## Conclusions

In this study we have expanded the utility of PAR-CLIP by developing a method to quantify background binding. We have shown that PAR-CLIP generates readily detectable, T-to-C containing background reads, and that PAR-CLIP data still contains background reads in spite of the assumption of detecting only direct protein-RNA interaction. We have also shown that background reads are reproducibly common in PAR-CLIP libraries, often with identical sequences appearing in libraries from many different RBPs and from experiments performed in several different laboratories. Despite the rigorous and stringent purification conditions of the PAR-CLIP procedure, background reads are easily detectable due to the extreme sensitivity of high-throughput sequencing [47]. Moreover, it should be noted that in none of the CLIP or PAR-CLIP studies has there been a formal demonstration that a covalent chemical crosslink has actually formed; rather, an operational assumption of crosslinking is asserted based upon survival after stringent washing. Taken together, these observations suggest that investigators using any crosslinking procedures should be cautious about the presence of background, since low efficiency conversion of a set of noncovalent bonds to a single covalent bond is not a guarantee of perfection, even when combined with rigorous and stringent purification conditions.

### Reproducible background reads and replicates

Background reads are found nearly universally across PAR-CLIP experiments from different RBPs for many sites and are often reproducible among background samples of different molecular weights, thus demonstrating that these background reads are an inherent characteristic of the process. This implies that if one only addresses non-specific binding by requiring reproducibility in replicates one will incorrectly regard these common and reproducible background reads as positives. However, requiring reproducibility in replicate samples is likely better at removing other background sources resulting from random events than using only quantitative measurement of background. Therefore, we recommend a combined approach of empirical background measurement and biological replicates to optimally minimize contribution of non-specific background binding events.

### PAR-CLIP background can derive from multiple sources

While the precise source of these background reads are unknown, it is likely that at least some of the reads containing T-to-C conversions are from non-specific protein-RNA interactions. These may arise in two categories: 1) RNAs that are not specifically recognized by the protein of interest, but that during the course of crosslinking are randomly proximal to reactive amino acids of the RBP of interest and become covalently linked; or 2) RNPs that do not contain the RBP of interest, yet are not entirely removed during the procedure. Non-specific protein-RNA interactions of the first type would not be expected to produce sites with much read depth or reproducibility because the interactions are random and must be physically juxtaposed to be crosslinked. Some of these non-specific interactions may include sites with one read that are uniquely present in gel slices. Of the second variety of non-specific protein-RNA interactions, these should be more numerous and more reproducible across experiments, and thus, are likely represented by some of the more abundant RNA sites we observed. Interestingly, these sites with deeper reads were frequently found in multiple background gel slices suggesting that they were not focused in any single location on the gel. There are several possible interpretations for this outcome; first gels may be overloaded in PAR-CLIP experiments so that they do not properly resolve the most abundant complexes. Secondly, these may represent sites with variable RNP composition, and thus, migrate at multiple locations on the gel. Additionally, there are still many other potential sources of background in crosslinking procedures that may or may not account for the high rate of T-to-C conversions. For example, RNAs can fold into conformations that can increase their “stickiness” or even potentially mimic protein epitopes [48]. Thus, these aptameric RNAs can be greatly enriched in background reads as both non-specific, sticky RNAs or as off-target RNAs such as epitope mimics.

### Radiochemical and radiophysical effects on nucleic acids

Alternatively, it is possible that damage caused by UV irradiation causes changes in migration patterns of nucleic acids as is known to occur in comet assays used to measure DNA damage [49]. In this scenario, electrophoresis may be less successful at focusing and separating signal RNPs from background RNPs, potentially leading to the detection of the same background reads in all three gel slices. On the other hand, irradiation induced damage is much less likely for PAR-CLIP which irradiates at 365 nm than for other crosslinking methods that use UV 254 nm, which is know to cause damage to nucleic acids and to affect translation or to generate cellular RNP aggregates [50]. Finally, it is possible that some of the abundant and reproducible background T-to-C conversions may not actually represent non-specific protein-RNA interactions but may instead be non-crosslinked, 4SU-containing RNA that still converts to cytosine following cDNA synthesis [18].

### Background binding is characteristic of all biochemical enrichments

Of the many potential background sources mentioned above, it is likely that they are not mutually exclusive and any combination, as well as any number of other unforeseen ones, could contribute to the measured background reads. The numerous and diverse sources of potential background combined with the extreme sensitivity of high-throughput sequencing demonstrate the impracticality of attempting to entirely eliminate background biochemically. Furthermore, inherent limitations of the procedures may prevent ideal optimization, as does the fact that the mRNAs in any given subset from an IP are likely to have a range of crosslinked efficiencies [21]. For example, trying to combat potential over-loading of gels by using less starting material or splitting samples over more wells may help lower background, but given the low efficiency of crosslinking, especially of UV 254 nm, this may reduce signal below acceptable levels [18]. Instead of attempting to optimize the elimination of biochemical background, we offer a more practical solution of empirically measuring background binding events in every experiment. We show that this quantified background can be used to correct for non-signal or potentially erroneous events in PAR-CLIP, thus sidestepping the impractical task of identifying all sources of background and reducing the optimization to reasonable and achievable levels.

In addition to being a practical solution, empirically measuring background can also offer substantial benefits, as seen with the improved detection of the Pum motifs and discovering novel regulatory elements such as the A-rich motif for Caprin1. The amount of improvement provided by background correction will likely vary depending on the RBP and the fastidious quality of the experiment. Given the large percent overlap of weak or under sequenced RBPs with background (up to 45%), this background correction will be especially important for defining and validating RNA targets of weak binding RBPs, RBP mutants or candidate novel RBPs identified by global approaches [21,38,39,51-55].

Interestingly, a recent global crosslinking study identified numerous RNAs associated with Ago2 in Dicer null cells despite the fact that the cells were lacking processed miRNAs [56]. These Ago2-associated RNAs were enriched for a G-rich motif that the authors suggest indicates a preference for Ago2 to bind to G’s in the absence of processed miRNAs. However, background binding was not directly measured in that study as well. Given our observation of a highly similar G-rich motif in PAR-CLIP background reads, a potential alternative explanation is that Ago2 doesn’t bind RNA in Dicer^-/-^ cells and that the G-rich RNAs simply represent background binding.

### Background measurements for global RNA dynamics studies with PAR-CLIP

The quantitative measurement of background in PAR-CLIP may also have benefits beyond improving percent signal and removing misleading results, it may also be used as a reference in determining fold enrichments, affinities for different RNAs or fold changes in binding during changes in cellular conditions, such as during immune activation. In these cases, measured background may prove more useful than adjusting for the level of expressed RNA because it will naturally incorporate the inherent biases of the procedure. This approach of using background as a reference has already been shown to be useful for detecting dynamic changes in RNP association during T-cell activation when applied to RIP-Chip [14]. This approach may prove especially beneficial when combined with computational approaches that model the quantitatively discrete nature of sequencing data [57].

### Matching background controls to experiments

We have shown that applying background correction to several published PAR-CLIP data sets of various RBPs can substantially improve results. This is surprising considering that these data sets were generated in different laboratories using different RBPs that migrate at different sizes. The reproducibility and universality of background reads across each of these independent experiments suggest that the background data we generated here may prove useful in future PAR-CLIP studies. However, it should be cautioned that with a few exceptions, the studies compared in this manuscript were from HEK293 lysates and the protocols were carried out with nearly identical conditions. Therefore, it remains to be seen whether the universality of these background reads hold up for PAR-CLIPs performed with different lysates or modifications to the protocol. This becomes especially obvious when considering cell-type-specific or condition-specific transcripts that would be present in experimental samples but not in the background samples preformed in this manuscript. One extreme example of condition-specific transcripts is Ago PAR-CLIP performed on HIV infected cells, where it was recently reported that Ago PAR-CLIP showed evidence of binding to a miR-29a site in the HIV genome but that this site was shown to be non-functional using RIP and reporter assays [58]. Since our background study was performed in cells that were not HIV infected, we have no way of knowing whether this site represents background binding or is an Ago-bound, non-functional site. Therefore, we would recommend performing and sequencing appropriate background controls matched with individual experiments whenever possible. We also recommend validation of targets through other methods including assays for functional responses.

### Future considerations

Much in the way PAR-CLIP has improved on UV 254 crosslinking methods by measuring T-to-C conversions we have improved upon PAR-CLIP by accounting for crosslinked background. We achieved this by borrowing an approach from RIP-Chip, namely, measuring background empirically. This approach is a key feature of RIP-Chip that allows for quantitative measurement of protein-RNA interactions, and more importantly, to compare RIP-Chip data from different conditions to determine protein-RNA dynamics. For PAR-CLIP and other global crosslinking techniques to reliably achieve these quantitative and dynamic measurements several considerations will still need to be addressed. One such issue is the presence of PCR amplification artifacts that can limit the quantitative analysis of protein-RNA interactions. This issue has already been addressed in several related crosslinking methods by the introduction of “randomer” barcodes into the library making process [15,59,60]. Another consideration is whether high-throughput sequencing of binding sites has reached saturation, and thus, whether sequencing depth is in the dynamic range for quantification of all binding sites. We are unaware of any global protein-RNA sequencing studies to date that have demonstrated full saturation. In the present study we failed to reach saturation despite pushing the current limits of sequencing technologies with approximately 250 million raw reads per library (Additional file 71: Figure S8). Future developments and refinements to global protein-RNA interaction studies, like PAR-CLIP, will lay the foundation for unraveling the “RNP code” and understanding the organizational and mechanistic properties underlying the dynamics of posttranscriptional RNA operons and regulons.

## Materials and methods

### Cell culture

Human embryonic kidney 293 cells (HEK 293) stably expressing Dox-inducible HuR or GFP were plated at 7.5 × 10^5 cells/ml and grown for 24 hours in normal growth media (DMEM with 10% Tet-reduced FBS). The cells were then grown overnight in media supplemented with 100 uM 4SU and 1 uM Doxycycline.

### PAR-CLIP

Procedure is similar to Hafner et al, with minor adjustments. Specifically, both RNase digestion steps used less RNase T1. In the first digestion step a final concentration of 0.5 U/ul of RNase T1 was used and in the second digestion step, the one after immunoprecipitation, a final concentration of 0.005 U/ul RNase T1 was used.

### Mapping, processing and analysis of sequencing data

50 bp single read libraries were run with a single sample per lane on the Illumina HiSeq 2000 instrument. Adapter sequences were removed, and reads containing fewer than 10 bp, were eliminated from further analysis. Reads were then mapped to the human genome (hg19) using bowtie with parameters suggested for use with PARalyzer, no more than 2 mismatches and 10 multi-mappers (-v 2 –m 10 --all --best --strata) [61]. Bowtie output was then further refined using the PARalyzer algorithm using standard parameters which restricted reads to only those containing 0, 1 or 2 T-to-C mismatches mapping uniquely to the genome [31]. PARalyzer “groups” were defined as sites with any read evidence and “clusters” were defined as sites with at least 2 T-to-C conversion locations and at least 5 overlapping reads.

### Motif analysis

*K-mer* length motifs were generated by quantifying the occurrence of each oligomer of length *k* in all PARalyzer utilized reads for a given library. The abundance of each k-mer was counted and rank ordered. Caprin1 motifs were identified by grouping similar sequences in the matched pairs analysis followed by alignment of the sequences by PhyloGibbs analysis. For background motifs the top 25 8-mers were used to make motif logos using enoLOGOS [62]. Significance of enrichment for motifs was determined by comparing observed frequencies to expected from shuffling the libraries while preserving di-nucleotide frequencies.

### Background correction

To remove reads from PAR-CLIP RBP libraries that were also present in the union of PAR-CLIP backgrounds, entire sites were removed if they overlapped by one or more bp between both libraries by using BEDTools [63].

### Saturation analysis

Saturation analysis was performed by randomly sampling reads prior to processing. 5 independent sets were sampled to depths to match 10, 30, 50, 70 and 90% of all reads. These sampled sets were then processed and analyzed as described above. The number of sites or clusters (defined as sites with 5 or more reads and two or more T-to-C conversion locations) for each sampled set were counted and reported as a fraction of all sites (or clusters) identified in the whole, un-sampled set.

### Data accessibility

Raw and processed data for the background data sets (G45, G35 and G20), two HuR replicates and total crosslinked RNA samples have been submitted to GEO (Accession number: GSE50989).

## Competing interests

J.D.K. has financial relationships with Ribonomics, Inc. and MBL, Inc. that hold licenses related to technologies used in this study. M.B.F. has no competing interests.

## Authors’ contributions

MBF performed experiments and analysis. MBF and JDK conceived of the study, designed the experiments, interpreted the results and prepared the manuscript. Both authors read and approved the final manuscript.

## Supplementary Material

Additional file 1 Is a figure of the mutational profile of bowtie mapped reads. Click here for file

Additional file 2 Is a figure showing correlations of background and HuR samples. Click here for file

Additional file 3 Is a figure showing background and HuR binding to full length MALAT1 transcript. Click here for file

Additional file 4 Is a figure showing background and HuR binding to full length ELAVL1 transcript. Click here for file

Additional file 5 **Is a table of re-analyzed PARalyzer output for clusters of at least 5 reads and 2 T-to-C conversions for the AGO2.EF3D library from **[64]**.** Click here for file

Additional file 6 **Is a table of re-analyzed PARalyzer output for groups indicating all sites utilized by PARalyzer for the AGO2.EF3D library from **[64]**.** Click here for file

Additional file 7 **Is a table of re-analyzed PARalyzer output for clusters of at least 5 reads and 2 T-to-C conversions for the AGO2.LCL35 library from **[64]**.** Click here for file

Additional file 8 **Is a table of re-analyzed PARalyzer output for groups indicating all sites utilized by PARalyzer for the AGO2.LCL35 library from **[64]**.** Click here for file

Additional file 9 **Is a table of re-analyzed PARalyzer output for clusters of at least 5 reads and 2 T-to-C conversions for the AGO2.LCLBAC library from **[64]**.** Click here for file

Additional file 10 **Is a table of re-analyzed PARalyzer output for groups indicating all sites utilized by PARalyzer for the AGO2.LCLBAC library from **[64]**.** Click here for file

Additional file 11 **Is a table of re-analyzed PARalyzer output for clusters of at least 5 reads and 2 T-to-C conversions for the AGO2.LCLBACD1 library from **[64]**.** Click here for file

Additional file 12 **Is a table of re-analyzed PARalyzer output for groups indicating all sites utilized by PARalyzer for the AGO2.LCLBACD1 library from **[64]**.** Click here for file

Additional file 13 **Is a table of re-analyzed PARalyzer output for clusters of at least 5 reads and 2 T-to-C conversions for the AGO2.BACD3 library from **[64]**.** Click here for file

Additional file 14 **Is a table of re-analyzed PARalyzer output for groups indicating all sites utilized by PARalyzer for the AGO2.BACD3 library from **[64]**.** Click here for file

Additional file 15 **Is a table of re-analyzed PARalyzer output for clusters of at least 5 reads and 2 T-to-C conversions for the AGO2.rep1 library from **[18]**.** Click here for file

Additional file 16 **Is a table of re-analyzed PARalyzer output for groups indicating all sites utilized by PARalyzer for the AGO2.rep1 library from **[18]**.** Click here for file

Additional file 17 **Is a table of re-analyzed PARalyzer output for clusters of at least 5 reads and 2 T-to-C conversions for the AGO2.rep2 library from **[18]**.** Click here for file

Additional file 18 **Is a table of re-analyzed PARalyzer output for groups indicating all sites utilized by PARalyzer for the AGO2.rep2 library from **[18]**.** Click here for file

Additional file 19 **Is a table of re-analyzed PARalyzer output for clusters of at least 5 reads and 2 T-to-C conversions for the ALKBH5.rep1 library from **[38]**.** Click here for file

Additional file 20 **Is a table of re-analyzed PARalyzer output for groups indicating all sites utilized by PARalyzer for the ALKBH5.rep1 library from **[38]**.** Click here for file

Additional file 21 **Is a table of re-analyzed PARalyzer output for clusters of at least 5 reads and 2 T-to-C conversions for the ALKBH5.rep2 library from **[38]**.** Click here for file

Additional file 22 **Is a table of re-analyzed PARalyzer output for groups indicating all sites utilized by PARalyzer for the ALKBH5.rep2 library from **[38]**.** Click here for file

Additional file 23 **Is a table of re-analyzed PARalyzer output for clusters of at least 5 reads and 2 T-to-C conversions for the C17ORF85.rep1 library from **[38]**.** Click here for file

Additional file 24 **Is a table of re-analyzed PARalyzer output for groups indicating all sites utilized by PARalyzer for the C17ORF85.rep1 library from **[38]**.** Click here for file

Additional file 25 **Is a table of re-analyzed PARalyzer output for clusters of at least 5 reads and 2 T-to-C conversions for the C17ORF85.rep2 library from **[38]**.** Click here for file

Additional file 26 **Is a table of re-analyzed PARalyzer output for groups indicating all sites utilized by PARalyzer for the C17ORF85.rep2 library from **[38]**.** Click here for file

Additional file 27 **Is a table of re-analyzed PARalyzer output for clusters of at least 5 reads and 2 T-to-C conversions for the C22ORF28.rep1 library from **[38]**.** Click here for file

Additional file 28 **Is a table of re-analyzed PARalyzer output for groups indicating all sites utilized by PARalyzer for the C22ORF28.rep1 library from **[38]**.** Click here for file

Additional file 29 **Is a table of re-analyzed PARalyzer output for clusters of at least 5 reads and 2 T-to-C conversions for the C22ORF28.rep2 library from **[38]**.** Click here for file

Additional file 30 **Is a table of re-analyzed PARalyzer output for groups indicating all sites utilized by PARalyzer for the C22ORF28.rep2 library from **[38]**.** Click here for file

Additional file 31 **Is a table of re-analyzed PARalyzer output for clusters of at least 5 reads and 2 T-to-C conversions for the CAPRIN1.rep1 library from **[38]**.** Click here for file

Additional file 32 **Is a table of re-analyzed PARalyzer output for groups indicating all sites utilized by PARalyzer for the CAPRIN1.rep1 library from **[38]**.** Click here for file

Additional file 33 **Is a table of re-analyzed PARalyzer output for clusters of at least 5 reads and 2 T-to-C conversions for the CAPRIN1.rep2 library from **[38]**.** Click here for file

Additional file 34 **Is a table of re-analyzed PARalyzer output for groups indicating all sites utilized by PARalyzer for the CAPRIN1.rep2 library from **[38]**.** Click here for file

Additional file 35 **Is a table of re-analyzed PARalyzer output for clusters of at least 5 reads and 2 T-to-C conversions for the FMR1.I304N.iso1 library from **[34]**.** Click here for file

Additional file 36 **Is a table of re-analyzed PARalyzer output for clusters of at least 5 reads and 2 T-to-C conversions for the FMR1.I304N.iso7 library from **[34]**.** Click here for file

Additional file 37 **Is a table of re-analyzed PARalyzer output for clusters of at least 5 reads and 2 T-to-C conversions for the FMR1.iso1 library from **[34]**.** Click here for file

Additional file 38 **Is a table of re-analyzed PARalyzer output for groups indicating all sites utilized by PARalyzer for the FMR.iso1 library from **[34]**.** Click here for file

Additional file 39 **Is a table of re-analyzed PARalyzer output for clusters of at least 5 reads and 2 T-to-C conversions for the FMR1.iso7 library from **[34]**.** Click here for file

Additional file 40 **Is a table of re-analyzed PARalyzer output for groups indicating all sites utilized by PARalyzer for the FMR.iso7 library from **[34]**.** Click here for file

Additional file 41 **Is a table of re-analyzed PARalyzer output for clusters of at least 5 reads and 2 T-to-C conversions for the FXR1 library from **[34]**.** Click here for file

Additional file 42 **Is a table of re-analyzed PARalyzer output for groups indicating all sites utilized by PARalyzer for the FXR1 library from **[34]**.** Click here for file

Additional file 43 **Is a table of re-analyzed PARalyzer output for clusters of at least 5 reads and 2 T-to-C conversions for the FXR2 library from **[34]**.** Click here for file

Additional file 44 **Is a table of re-analyzed PARalyzer output for groups indicating all sites utilized by PARalyzer for the FXR2 library from **[34]**.** Click here for file

Additional file 45 **Is a table of re-analyzed PARalyzer output for clusters of at least 5 reads and 2 T-to-C conversions for the IGF2BP2.rep1 library from **[18]**.** Click here for file

Additional file 46 **Is a table of re-analyzed PARalyzer output for groups indicating all sites utilized by PARalyzer for the IGF2BP2.rep1 library from **[18]**.** Click here for file

Additional file 47 **Is a table of re-analyzed PARalyzer output for clusters of at least 5 reads and 2 T-to-C conversions for the IGF2BP2.rep2 library from **[18]**.** Click here for file

Additional file 48 **Is a table of re-analyzed PARalyzer output for groups indicating all sites utilized by PARalyzer for the IGF2BP2.rep2 library from **[18]**.** Click here for file

Additional file 49 **Is a table of re-analyzed PARalyzer output for clusters of at least 5 reads and 2 T-to-C conversions for the IGF2BP2.rep3 library from **[18]**.** Click here for file

Additional file 50 **Is a table of re-analyzed PARalyzer output for groups indicating all sites utilized by PARalyzer for the IGF2BP2.rep3 library from **[18]**.** Click here for file

Additional file 51 **Is a table of re-analyzed PARalyzer output for clusters of at least 5 reads and 2 T-to-C conversions for the IGF2BP2.rep4 library from **[18]**.** Click here for file

Additional file 52 **Is a table of re-analyzed PARalyzer output for groups indicating all sites utilized by PARalyzer for the IGF2BP2.rep4 library from **[18]**.** Click here for file

Additional file 53 **Is a table of re-analyzed PARalyzer output for clusters of at least 5 reads and 2 T-to-C conversions for the IGF2BP2.rep5 library from **[18]**.** Click here for file

Additional file 54 **Is a table of re-analyzed PARalyzer output for groups indicating all sites utilized by PARalyzer for the IGF2BP2.rep5 library from **[18]**.** Click here for file

Additional file 55 **Is a table of re-analyzed PARalyzer output for clusters of at least 5 reads and 2 T-to-C conversions for the HuR.DMEM library from **[37]**.** Click here for file

Additional file 56 **Is a table of re-analyzed PARalyzer output for groups indicating all sites utilized by PARalyzer for the HuR.DMEM library from **[37]**.** Click here for file

Additional file 57 **Is a table of re-analyzed PARalyzer output for clusters of at least 5 reads and 2 T-to-C conversions for the HuR.SILAC library from **[37]**.** Click here for file

Additional file 58 **Is a table of re-analyzed PARalyzer output for groups indicating all sites utilized by PARalyzer for the HuR.SILAC library from **[37]**.** Click here for file

Additional file 59 **Is a table of re-analyzed PARalyzer output for clusters of at least 5 reads and 2 T-to-C conversions for the PUM2.rep1 library from **[18]**.** Click here for file

Additional file 60 **Is a table of re-analyzed PARalyzer output for groups indicating all sites utilized by PARalyzer for the PUM2.rep1 library from **[18]**.** Click here for file

Additional file 61 **Is a table of re-analyzed PARalyzer output for clusters of at least 5 reads and 2 T-to-C conversions for the PUM2.rep2 library from **[18]**.** Click here for file

Additional file 62 **Is a table of re-analyzed PARalyzer output for groups indicating all sites utilized by PARalyzer for the PUM2.rep2 library from **[18]**.** Click here for file

Additional file 63 **Is a table of re-analyzed PARalyzer output for clusters of at least 5 reads and 2 T-to-C conversions for the ZC3H7B.rep1 library from **[38]**.** Click here for file

Additional file 64 **Is a table of re-analyzed PARalyzer output for groups indicating all sites utilized by PARalyzer for the ZC3H7B.rep1 library from **[38]**.** Click here for file

Additional file 65 **Is a table of re-analyzed PARalyzer output for clusters of at least 5 reads and 2 T-to-C conversions for the ZC3H7B.rep2 library from **[38]**.** Click here for file

Additional file 66 **Is a table of re-analyzed PARalyzer output for groups indicating all sites utilized by PARalyzer for the ZC3H7B.rep3 library from **[38]**.** Click here for file

Additional file 67 Is a figure showing binding from multiple PAR-CLIP libraries of different RBPs for full length MALAT1 transcript. Click here for file

Additional file 68 **Contains a table listing details of the libraries used in analysis for Figure** 5**B, as well as Supplemental Figure legends and Supplemental References.** Click here for file

Additional file 69 Is a figure showing percent overlap of high abundance sites with background sites. Click here for file

Additional file 70 Is a figure showing background correction of Caprin1 PAR-CLIP enriches for A-rich motifs versus U-rich motifs. Click here for file

Additional file 71 Is a figure showing saturation analysis of PAR-CLIP libraries. Click here for file
