# Supplementary figures and images for: Advancing the functional utility of PAR-CLIP by quantifying background binding to mRNAs and lncRNAs
Source: Genome Biol. 2014 Jan 7;15(1):R2. doi: 10.1186/gb-2014-15-1-r2 (PMC4053780; doi:10.1186/gb-2014-15-1-r2)

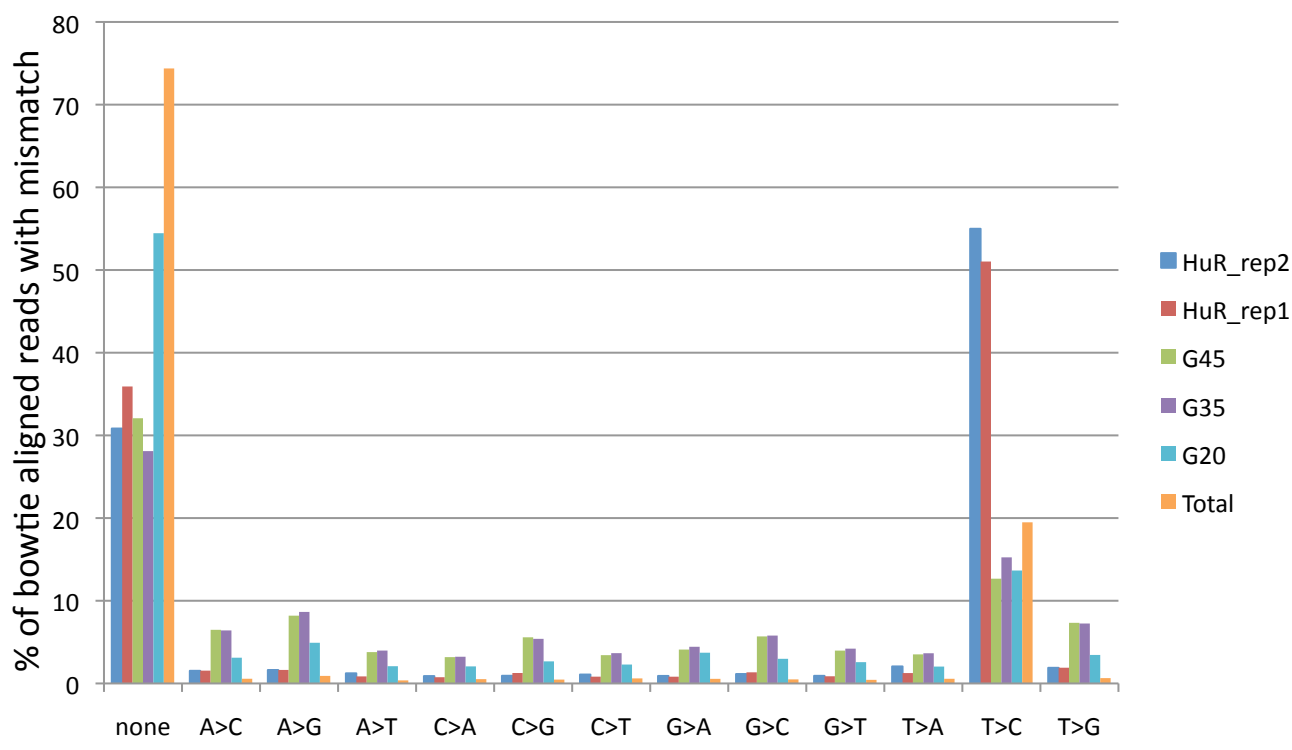

Supplement: Additional file 1 — Is a figure of the mutational profile of bowtie mapped reads. [file gb-2014-15-1-r2-S1.pdf]

# MALAT1 (chr11:65,265,000-65,274,100)

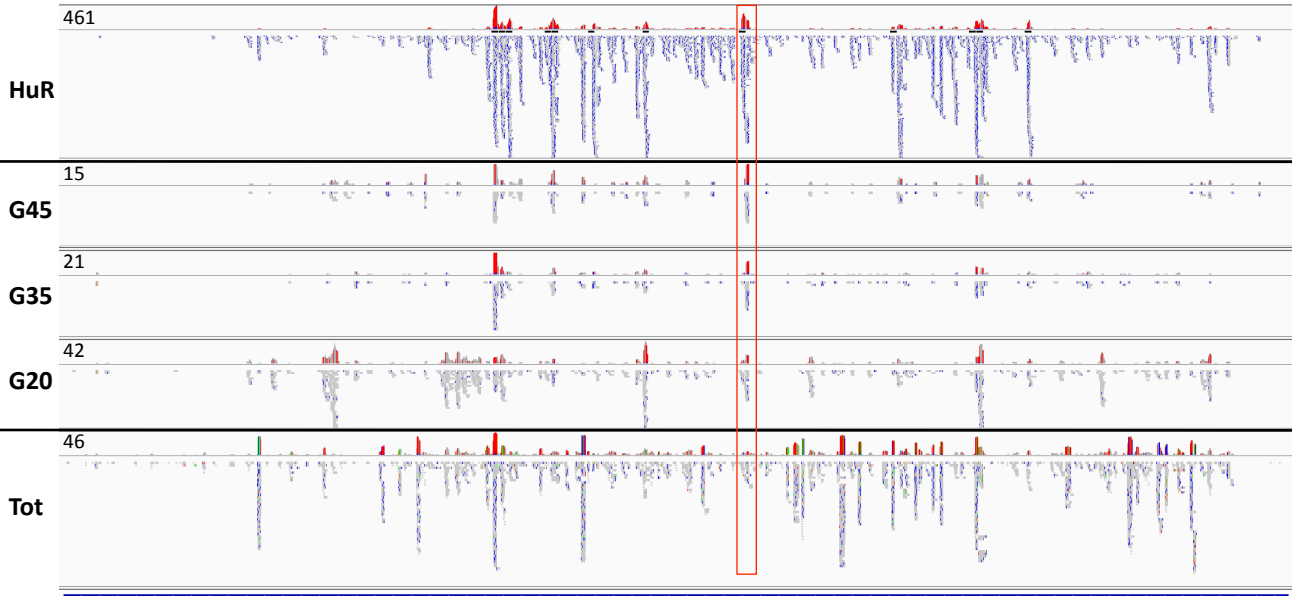

Supplement: Additional file 3 — Is a figure showing background and HuR binding to full length MALAT1 transcript. [file gb-2014-15-1-r2-S3.pdf]

# Full length **ELAVL1** (chr19:8,023,000-8,070,800)

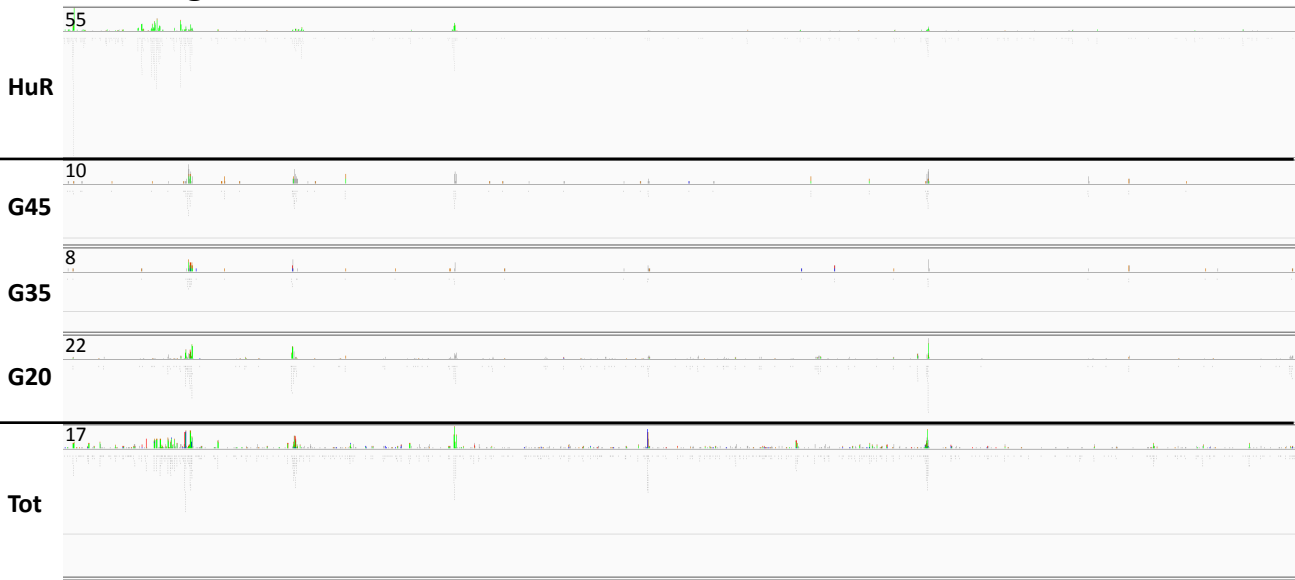

## Last exon **ELAVL1** (chr19:8,023,000-8,028,800)

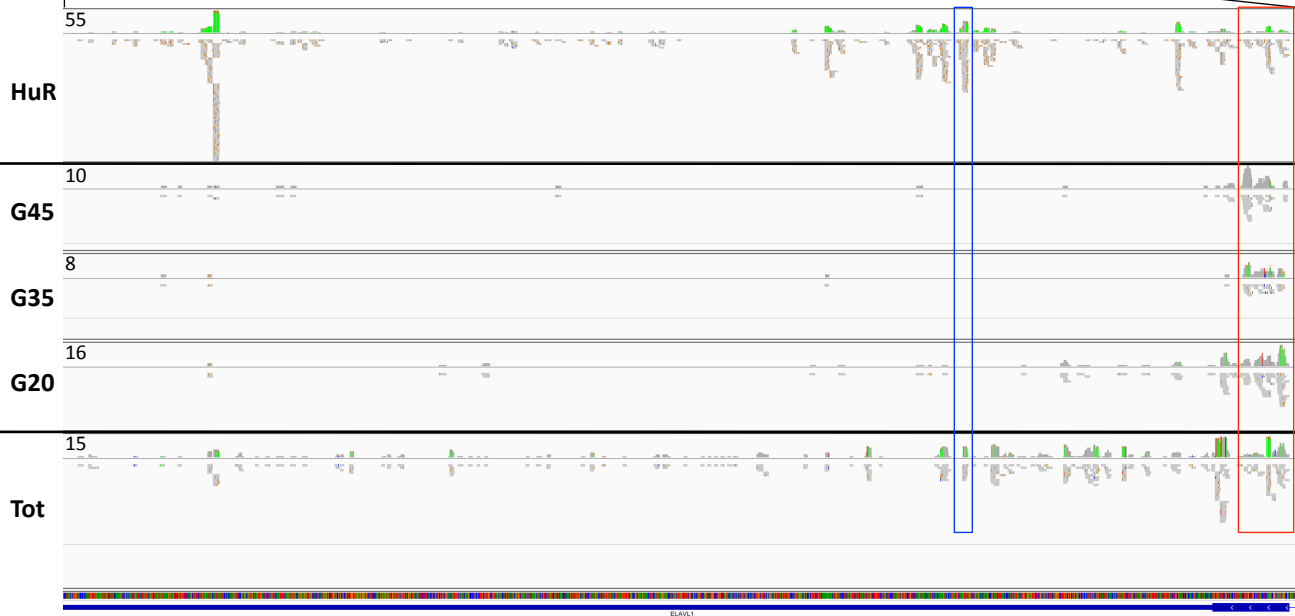

Supplement: Additional file 4 — Is a figure showing background and HuR binding to full length ELAVL1 transcript. [file gb-2014-15-1-r2-S4.pdf]

fraction overlap with background

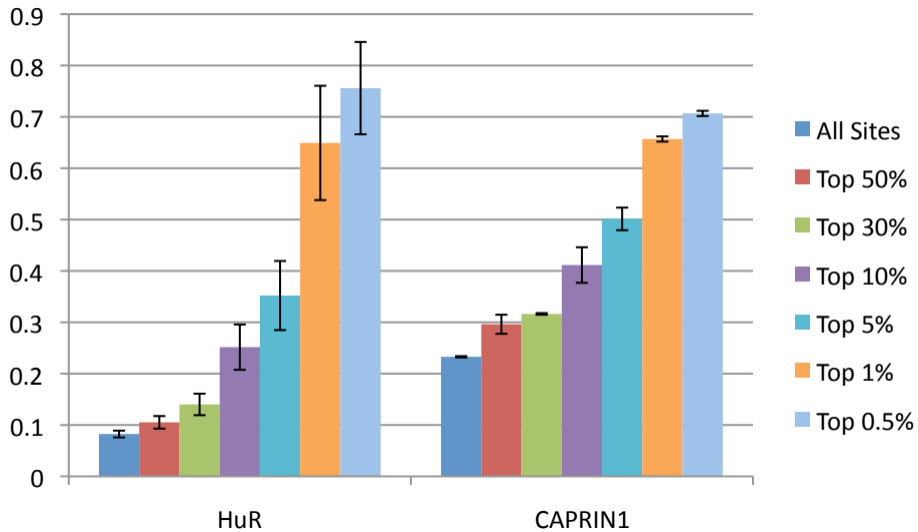

Supplement: Additional file 69 — Is a figure showing percent overlap of high abundance sites with background sites. [file gb-2014-15-1-r2-S69.pdf]

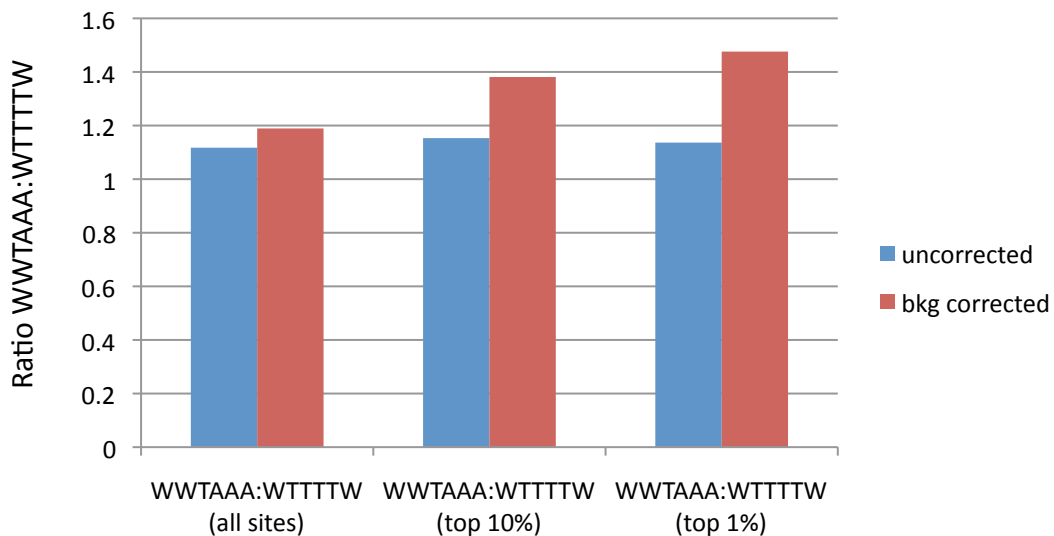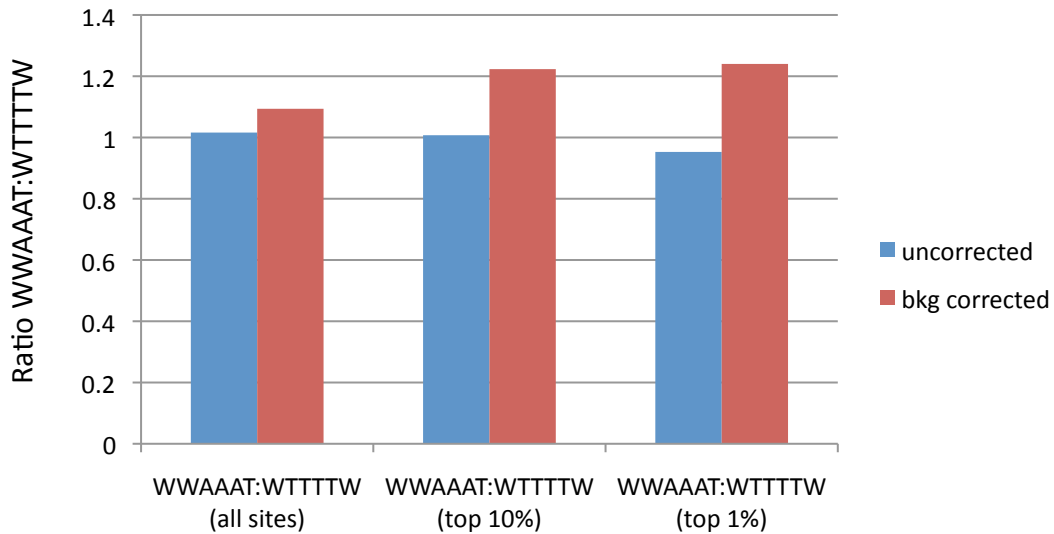

Supplement: Additional file 70 — Is a figure showing background correction of Caprin1 PAR-CLIP enriches for A-rich motifs versus U-rich motifs. [file gb-2014-15-1-r2-S70.pdf]

# PARalyzer cluster reads

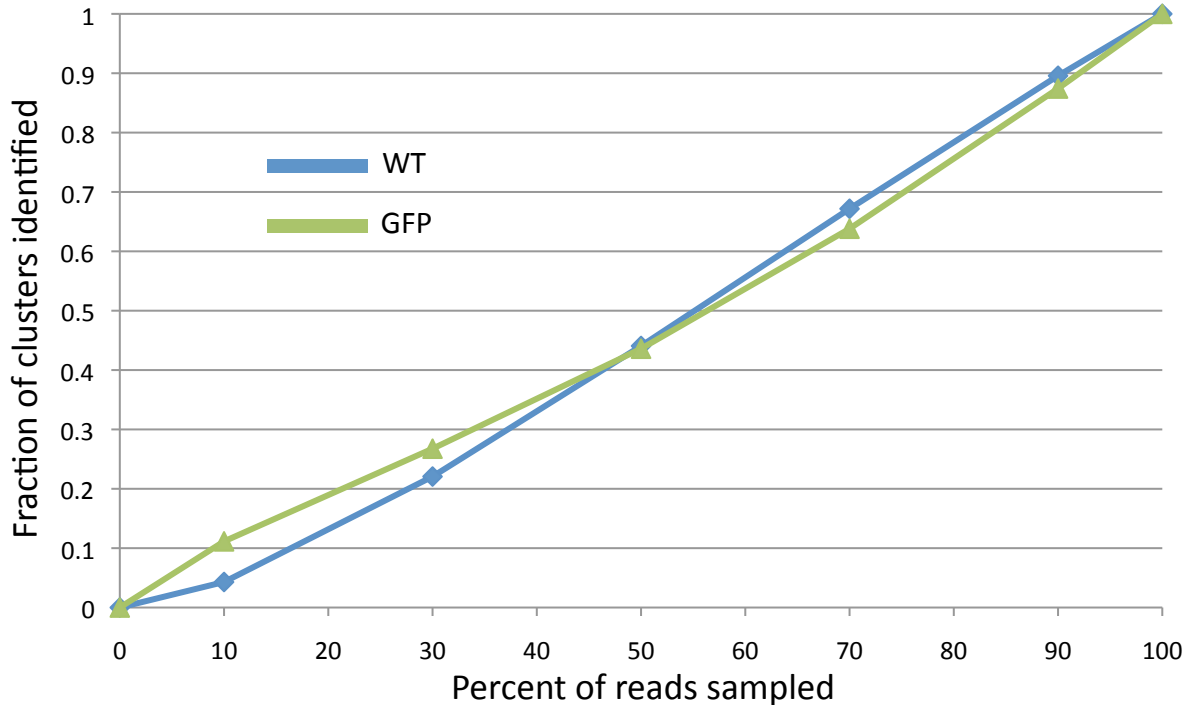

Supplement: Additional file 71 — Is a figure showing saturation analysis of PAR-CLIP libraries. [file gb-2014-15-1-r2-S71.pdf]
