# Supplementary material for: Advancing the functional utility of PAR-CLIP by quantifying background binding to mRNAs and lncRNAs
Source: Genome Biol. 2014 Jan 7;15(1):R2. doi: 10.1186/gb-2014-15-1-r2 (PMC4053780; doi:10.1186/gb-2014-15-1-r2)

A)

Multivariate

Correlations

	log(G45 reads)	log(G35 reads)	log(G20 reads)
log(G45 reads)	1.0000	0.6954	0.6483
log(G35 reads)	0.6954	1.0000	0.6725
log(G20 reads)	0.6483	0.6725	1.0000

Scatterplot Matrix

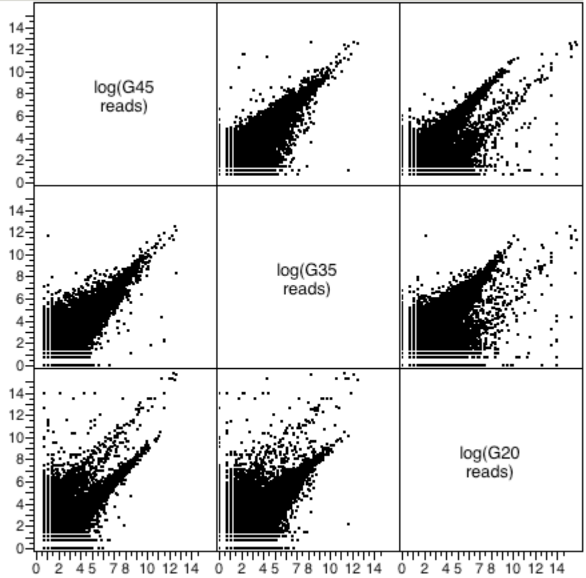

B)

Multivariate

Correlations

	log(union HuR reads)	log(union background reads)
log(union HuR reads)	1.0000	0.2961
log(union background reads)	0.2961	1.0000

Scatterplot Matrix

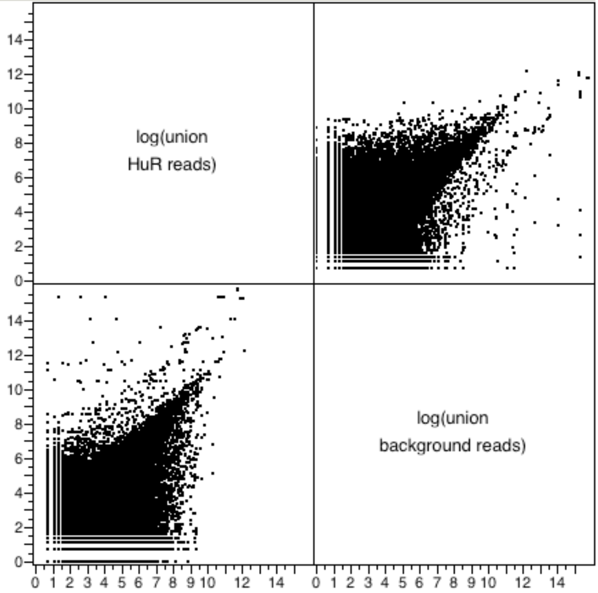

Supplement: Additional file 2 — Is a figure showing correlations of background and HuR samples. [file gb-2014-15-1-r2-S2.pdf]
