# Supplementary material for: Advancing the functional utility of PAR-CLIP by quantifying background binding to mRNAs and lncRNAs
Source: Genome Biol. 2014 Jan 7;15(1):R2. doi: 10.1186/gb-2014-15-1-r2 (PMC4053780; doi:10.1186/gb-2014-15-1-r2)
Supplement: Additional file 67 — Is a figure showing binding from multiple PAR-CLIP libraries of different RBPs for full length MALAT1 transcript. [file gb-2014-15-1-r2-S67.pdf]

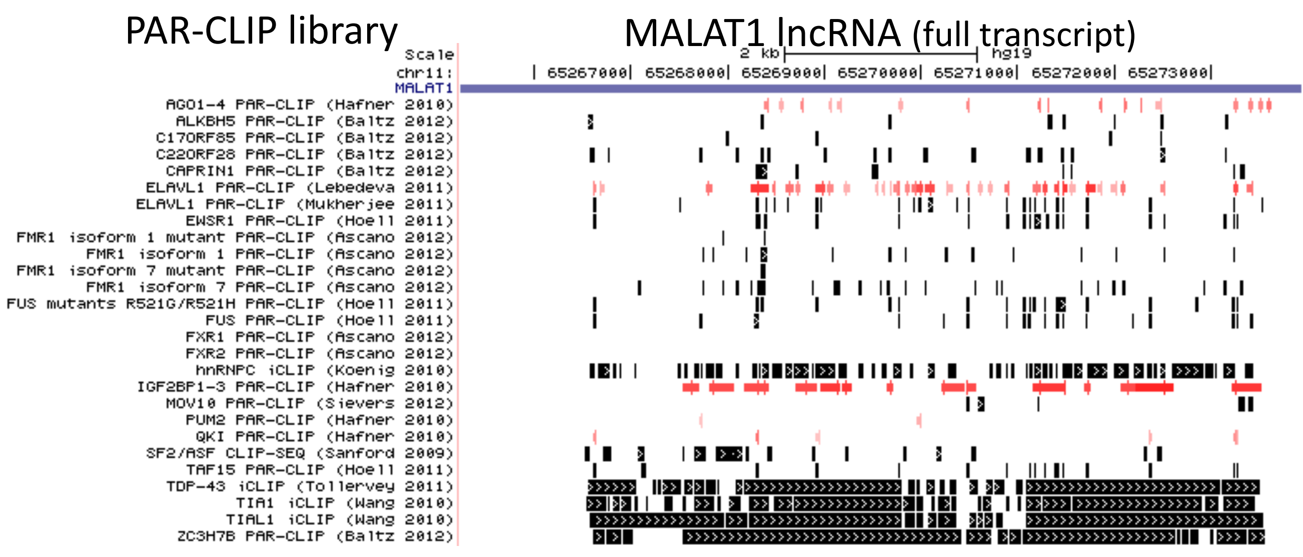
PAR-CLIP library
MALAT1 lncRNA (full transcript)
Scale
2 kb
hg19
chr11:
65267000
65268000
65269000
65270000
65271000
65272000
65273000
MALAT1
AGO1-4 PAR-CLIP (Hafner 2010)
ALKBH5 PAR-CLIP (Baltz 2012)
C17ORF85 PAR-CLIP (Baltz 2012)
C22ORF28 PAR-CLIP (Baltz 2012)
CAPRIN1 PAR-CLIP (Baltz 2012)
ELAVL1 PAR-CLIP (Lebedeva 2011)
ELAVL1 PAR-CLIP (Mukherjee 2011)
EWSR1 PAR-CLIP (Hoell 2011)
FMR1 isoform 1 mutant PAR-CLIP (Ascano 2012)
FMR1 isoform 1 PAR-CLIP (Ascano 2012)
FMR1 isoform 7 mutant PAR-CLIP (Ascano 2012)
FMR1 isoform 7 PAR-CLIP (Ascano 2012)
FUS mutants R521G/R521H PAR-CLIP (Hoell 2011)
FUS PAR-CLIP (Hoell 2011)
FXR1 PAR-CLIP (Ascano 2012)
FXR2 PAR-CLIP (Ascano 2012)
hnRNPC iCLIP (Koenig 2010)
IGF2BP1-3 PAR-CLIP (Hafner 2010)
MOV10 PAR-CLIP (Sievers 2012)
PUM2 PAR-CLIP (Hafner 2010)
QKI PAR-CLIP (Hafner 2010)
SF2/ASF CLIP-SEQ (Sanford 2009)
TAF15 PAR-CLIP (Hoell 2011)
TDP-43 iCLIP (Tollervey 2011)
TIA1 iCLIP (Wang 2010)
TIAL1 iCLIP (Wang 2010)
ZC3H7B PAR-CLIP (Baltz 2012)
