# Supplementary material for: Advancing the functional utility of PAR-CLIP by quantifying background binding to mRNAs and lncRNAs
Source: Genome Biol. 2014 Jan 7;15(1):R2. doi: 10.1186/gb-2014-15-1-r2 (PMC4053780; doi:10.1186/gb-2014-15-1-r2)
Supplement: Additional file 68 — Contains a table listing details of the libraries used in analysis for Figure 5B, as well as Supplemental Figure legends and Supplemental References. [file gb-2014-15-1-r2-S68.docx]

**Supplemental Table**

**Supplemental Table 1. PAR-CLIP library information for Figure 5B.** Table listing library identifier, RBP name (with replicate number, if applicable), citation, and accession number.

**Supplemental Table 1**

|  | **RBP** | **study** | **accession number** |
| --- | --- | --- | --- |
| lib1 | ALKBH5.rep1 | Baltz et al, 2012 [[1](#_ENREF_1)] | SRX149417 |
| lib2 | IGF2BP2.rep5 | Hafner et al, 2010 [[2](#_ENREF_2)] | SRX020779 |
| lib3 | Ago2.rep2 | Hafner et al, 2010 [[2](#_ENREF_2)] | SRX020784 |
| lib4 | C17ORF85.rep1 | Baltz et al, 2012 [[1](#_ENREF_1)] | SRX149418 |
| lib5 | Pum2.rep1 | Hafner et al, 2010 [[2](#_ENREF_2)] | SRX020781 |
| lib6 | AGO2.LCLBACD3 | Skalsky et al, 2012 [[3](#_ENREF_3)] | SRX195375 |
| lib7 | Ago2.rep1 | Hafner et al, 2010 [[2](#_ENREF_2)] | SRX020784 |
| lib8 | AGO2.LCLBACD1 | Skalsky et al, 2012 [[3](#_ENREF_3)] | SRX195374 |
| lib9 | C22ORF28.rep2 | Baltz et al, 2012 [[1](#_ENREF_1)] | SRX149419 |
| lib10 | IGF2BP2.rep3 | Hafner et al, 2010 [[2](#_ENREF_2)] | SRX020779 |
| lib11 | ALKBH5.rep2 | Baltz et al, 2012 [[1](#_ENREF_1)] | SRX149417 |
| lib12 | C22ORF28.rep1 | Baltz et al, 2012 [[1](#_ENREF_1)] | SRX149419 |
| lib13 | CAPRIN1.rep2 | Baltz et al, 2012 [[1](#_ENREF_1)] | SRX149420 |
| lib14 | IGF2BP2.rep4 | Hafner et al, 2010 [[2](#_ENREF_2)] | SRX020779 |
| lib15 | CAPRIN1.rep1 | Baltz et al, 2012 [[1](#_ENREF_1)] | SRX149420 |
| lib16 | IGF2BP2.rep2 | Hafner et al, 2010 [[2](#_ENREF_2)] | SRX020779 |
| lib17 | FMR1.iso7 | Ascano et al, 2012 [[4](#_ENREF_4)] | SRX171148 |
| lib18 | AGO2.BAC | Skalsky et al, 2012 [[3](#_ENREF_3)] | SRX195373 |
| lib19 | AGO2.LCL35 | Skalsky et al, 2012 [[3](#_ENREF_3)] | SRX195372 |
| lib20 | AGO2.EF3D | Skalsky et al, 2012 [[3](#_ENREF_3)] | SRX195371 |
| lib21 | Pum2.rep2 | Hafner et al, 2010 [[2](#_ENREF_2)] | SRX020781 |
| lib22 | FXR2 | Ascano et al, 2012 [[4](#_ENREF_4)] | SRX171152 |
| lib23 | FMR1.iso1 | Ascano et al, 2012 [[4](#_ENREF_4)] | SRX171147 |
| lib24 | HuR | Lebedeva et al, 2011 [[5](#_ENREF_5)] | SRX083306 |
| lib25 | FXR1 | Ascano et al, 2012 [[4](#_ENREF_4)] | SRX171151 |
| lib26 | IGF2BP2.rep1 | Hafner et al, 2010 [[2](#_ENREF_2)] | SRX020779 |
| lib27 | FMR1.I304N.iso1 | Ascano et al, 2012 [[4](#_ENREF_4)] | SRX171149 |
| lib28 | HuR | Mukherjee et al, 2011 [[6](#_ENREF_6)] | SRX072653 |
| lib29 | FMR1.I304N.iso7 | Ascano et al, 2012 [[4](#_ENREF_4)] | SRX171150 |
| lib30 | ZC3H7B.rep1 | Baltz et al, 2012 [[1](#_ENREF_1)] | SRX149421 |
| lib31 | HuR.rep1 | this study | GSE50989 |
| lib32 | ZC3H7B.rep2 | Baltz et al, 2012 [[1](#_ENREF_1)] | SRX149421 |
| lib33 | HuR.rep2 | this study | GSE50989 |

**Supplemental Figure Legends**

**Supplemental Figure 1. PAR-CLIP libraries include large percentages of T-to-C mismatches.** Mutational profile of uniquely mapping reads containing zero or 1 mismatch from the six original data sets in this study. ‘None’ indicates reads that aligned to the reference genome with no mismatches, others indicate reference base followed by ‘>’ and mismatch in read. Only ‘none’ and ‘T>C’ reads are utilized by PARalyzer.

**Supplemental Figure 2. Background samples correlate well with each other and HuR correlates moderately well with background.** Panels show scatterplot correlations of log transformed reads along with R^2^ values. A) Correlations of the three background samples (G20, G35, and G45) with each other. B) Correlations of the union of two HuR replicates with three background samples.

**Supplemental Figure 3. Alignment of reads for full length *MALAT1* transcript.** PARalyzer utilized reads for HuR, background samples (G45, G35, and G20), and total libraries are shown for the full length *MALAT1* transcript. Blue tick marks indicate positions of T-to-C conversions. Red box indicates region that was expanded for **Fig. 4** in main text.

**Supplemental Figure 4. Alignment of reads for full length E*LAVL1* transcript.** PARalyzer utilized reads for HuR, background samples (G45, G35, and G20), and total libraries are shown for the full length *ELAVL1* transcript and a zoomed in view of the last exon. Tan tick marks indicate positions of T-to-C conversions. Red box and blue box indicates coding region and 3’UTR region, respectively, that were expanded for **Fig. 4** in main text. Blue bar at bottom of figure indicates annotated regions of *ELAVL1*.

**Supplemental Figure 5. Sequencing reads from different PAR-CLIP and iCLIP experiments are common in full length *MALAT1* transcript**. Screen shot from the doRiNA database [[7](#_ENREF_7)] showing the reported sequencing reads from many different PAR-CLIP and iCLIP experiments. The blue bar at the top represents full length *MALAT1* transcript and red and black bars represent read evidence from the indicated PAR-CLIP and iCLIP experiments.

**Supplemental Figure 6. High abundant sites overlap with background sites with greater frequency than low abundance sites**. Bars indicate the fraction of sites overlapping with background for the indicated top percentage of sites ranked by total number of reads for HuR PAR-CLIP libraries (from this paper) and for Caprin1 PAR-CLIP studies (from Baltz and colleagues [[1](#_ENREF_1)]).

**Supplemental Figure 7. Background correction of Caprin1 PAR-CLIP enriches A-rich motif versus U-rich motif, while the enrichment is not conclusive in uncorrected samples**. Ratio of number of sites containing A-rich motifs (WWTAAA in top and WWAAAT in bottom panels) to number of sites containing U-rich motifs (WTTTTW). Blue bars indicate uncorrected ratios and red bars indicate background corrected ratios. Top percentages of enriched sites are ranked by total number of reads for Caprin1 libraries.

**Supplemental Figure 8. HuR and background PAR-CLIP libraries are not saturated.** Line graph depicting linear relationship between number of sequencing reads and number of unique biding sites (clusters) for HuR (blue) and background (green) libraries. Note that these profiles are similar to most other published PAR-CLIP studies.

**Supplemental References**

1. Baltz AG, Munschauer M, Schwanhausser B, Vasile A, Murakawa Y, Schueler M, Youngs N, Penfold-Brown D, Drew K, Milek M *et al*: **The mRNA-bound proteome and its global occupancy profile on protein-coding transcripts**. *Mol Cell* 2012, **46**(5):674-690.

2. Hafner M, Landthaler M, Burger L, Khorshid M, Hausser J, Berninger P, Rothballer A, Ascano M, Jr., Jungkamp AC, Munschauer M *et al*: **Transcriptome-wide identification of RNA-binding protein and microRNA target sites by PAR-CLIP**. *Cell* 2010, **141**(1):129-141.

3. Skalsky RL, Corcoran DL, Gottwein E, Frank CL, Kang D, Hafner M, Nusbaum JD, Feederle R, Delecluse HJ, Luftig MA *et al*: **The viral and cellular microRNA targetome in lymphoblastoid cell lines**. *PLoS Pathog* 2012, **8**(1):e1002484.

4. Ascano M, Jr., Mukherjee N, Bandaru P, Miller JB, Nusbaum JD, Corcoran DL, Langlois C, Munschauer M, Dewell S, Hafner M *et al*: **FMRP targets distinct mRNA sequence elements to regulate protein expression**. *Nature* 2012, **492**(7429):382-386.

5. Lebedeva S, Jens M, Theil K, Schwanhausser B, Selbach M, Landthaler M, Rajewsky N: **Transcriptome-wide analysis of regulatory interactions of the RNA-binding protein HuR**. *Mol Cell* 2011, **43**(3):340-352.

6. Mukherjee N, Corcoran DL, Nusbaum JD, Reid DW, Georgiev S, Hafner M, Ascano M, Jr., Tuschl T, Ohler U, Keene JD: **Integrative regulatory mapping indicates that the RNA-binding protein HuR couples pre-mRNA processing and mRNA stability**. *Mol Cell* 2011, **43**(3):327-339.

7. Anders G, Mackowiak SD, Jens M, Maaskola J, Kuntzagk A, Rajewsky N, Landthaler M, Dieterich C: **doRiNA: a database of RNA interactions in post-transcriptional regulation**. *Nucleic Acids Res* 2012, **40**(Database issue):D180-186.
